# Supplementary material for: COZOID: contact zone identifier for visual analysis of protein-protein interactions
Source: BMC Bioinformatics. 2018 Apr 6;19:125. doi: 10.1186/s12859-018-2113-6 (PMC5889581; doi:10.1186/s12859-018-2113-6)
Supplement: Supplementary file 2 — Software build. Executable binary file of the software tool. (ZIP 118,784 kb) [file 12859_2018_2113_MOESM2_ESM.zip › cozoid/java/docs/beansbinding-1.2.1-doc.zip!/index-files/index-7.html]

 
 
 
 
 
 
G-Index (Beans Binding)
 

 

 

 
function windowTitle()
{
    if (location.href.indexOf('is-external=true') == -1) {
        parent.document.title="G-Index (Beans Binding)";
    }
}
 
 
 

 

 
 


 
   
  
 
 
 
   
 
   
          Overview     
        Package   
        Class   
        Use   
          Tree     
          Deprecated     
       Index    
          Help     
   
 
 
  
 
 
 

 
  
   PREV LETTER   
   NEXT LETTER    
  
    FRAMES     
   NO FRAMES     
  
    All Classes  ');
  }
  //-->
 
 
    All Classes  
 


  
 
 
  
 

 A   B   C   D   E   F   G   H   I   J   K   L   M   N   O   P   Q   R   S   T   U   V   W   
    
 G  
 
   GE0   - 
Static variable in interface org.jdesktop.el.impl.parser. ELParserConstants 
  
   GE1   - 
Static variable in interface org.jdesktop.el.impl.parser. ELParserConstants 
  
   generateParseException()   - 
Method in class org.jdesktop.el.impl.parser. ELParser 
  
   get(String)   - 
Static method in class org.jdesktop.el.impl.util. MessageFactory 
  
   get(String, Object)   - 
Static method in class org.jdesktop.el.impl.util. MessageFactory 
  
   get(String, Object, Object)   - 
Static method in class org.jdesktop.el.impl.util. MessageFactory 
  
   get(String, Object, Object, Object)   - 
Static method in class org.jdesktop.el.impl.util. MessageFactory 
  
   get(String, Object, Object, Object, Object)   - 
Static method in class org.jdesktop.el.impl.util. MessageFactory 
  
   get(String, Object, Object, Object, Object, Object)   - 
Static method in class org.jdesktop.el.impl.util. MessageFactory 
  
   getAdapter(Object, String)   - 
Static method in class org.jdesktop.beansbinding.ext. BeanAdapterFactory 
  
   getAdapter0(Object, String)   - 
Method in class org.jdesktop.beansbinding.ext. BeanAdapterFactory 
  
   getAdapterClass(Class<?>)   - 
Method in interface org.jdesktop.beansbinding.ext. BeanAdapterProvider 
  
   getAdapterClass(Class<?>)   - 
Method in class org.jdesktop.swingbinding.adapters. AbstractButtonAdapterProvider 
  
   getAdapterClass(Class<?>)   - 
Method in class org.jdesktop.swingbinding.adapters. JComboBoxAdapterProvider 
  
   getAdapterClass(Class<?>)   - 
Method in class org.jdesktop.swingbinding.adapters. JListAdapterProvider 
  
   getAdapterClass(Class<?>)   - 
Method in class org.jdesktop.swingbinding.adapters. JSliderAdapterProvider 
  
   getAdapterClass(Class<?>)   - 
Method in class org.jdesktop.swingbinding.adapters. JSpinnerAdapterProvider 
  
   getAdapterClass(Class<?>)   - 
Method in class org.jdesktop.swingbinding.adapters. JTableAdapterProvider 
  
   getAdapterClass(Class<?>)   - 
Method in class org.jdesktop.swingbinding.adapters. JTextComponentAdapterProvider 
  
   getAdapterPropertyDescriptors(Class<?>)   - 
Static method in class org.jdesktop.beansbinding.ext. BeanAdapterFactory 
  
   getArray(String, Object[])   - 
Static method in class org.jdesktop.el.impl.util. MessageFactory 
  
   getBeanProperty(String)   - 
Method in class org.jdesktop.el. BeanELResolver.BeanProperties 
  
   getBeginColumn()   - 
Method in class org.jdesktop.el.impl.parser. SimpleCharStream 
  
   getBeginLine()   - 
Method in class org.jdesktop.el.impl.parser. SimpleCharStream 
  
   getBinding(String)   - 
Method in class org.jdesktop.beansbinding. BindingGroup 
 Returns the  Binding  in this group with the given name,
 or  null  if this group doesn't contain a  Binding 
 with the given name.
   getBindingListeners()   - 
Method in class org.jdesktop.beansbinding. Binding 
 Returns the list of  BindingListeners  registered on this
  Binding .
   getBindingListeners()   - 
Method in class org.jdesktop.beansbinding. BindingGroup 
 Returns the list of  BindingListeners  registered on this
 group.
   getBindings()   - 
Method in class org.jdesktop.beansbinding. BindingGroup 
 Returns a list of all  Bindings  in this group.
   getColBindings()   - 
Method in class org.jdesktop.swingbinding.impl. ListBindingManager 
  
   getColumn()   - 
Method in class org.jdesktop.el.impl.parser. SimpleCharStream 
  Deprecated.     
   getColumn()   - 
Method in class org.jdesktop.swingbinding.impl. AbstractColumnBinding 
  
   getColumnBinding(int)   - 
Method in class org.jdesktop.swingbinding. JTableBinding 
 Returns the  ColumnBinding  with the given index in the list maintained
 by this  JTableBinding .
   getColumnBindings()   - 
Method in class org.jdesktop.swingbinding. JTableBinding 
 Returns an unmodifiable copy of the list of  ColumnBindings  maintained
 by this  JTableBinding .
   getColumnClass()   - 
Method in class org.jdesktop.swingbinding. JTableBinding.ColumnBinding 
 Returns the column class to be used by  JTable  to determine
 the renderer and editor for the column represented by this
  ColumnBinding .
   getColumnName()   - 
Method in class org.jdesktop.swingbinding. JTableBinding.ColumnBinding 
 Returns the name for the column represented by this  ColumnBinding .
   getCommonPropertyType(ELContext, Object)   - 
Method in class org.jdesktop.el. ArrayELResolver 
 If the base object is a Java language array, returns the most general 
 type that this resolver accepts for the  property  argument.
   getCommonPropertyType(ELContext, Object)   - 
Method in class org.jdesktop.el. BeanELResolver 
 If the base object is not  null , returns the most 
 general type that this resolver accepts for the 
  property  argument.
   getCommonPropertyType(ELContext, Object)   - 
Method in class org.jdesktop.el. CompositeELResolver 
 Returns the most general type that this resolver accepts for the
  property  argument, given a  base  object.
   getCommonPropertyType(ELContext, Object)   - 
Method in class org.jdesktop.el. ELResolver 
 Returns the most general type that this resolver accepts for the
  property  argument, given a  base  object.
   getCommonPropertyType(ELContext, Object)   - 
Method in class org.jdesktop.el. ListELResolver 
 If the base object is a list, returns the most general type that 
 this resolver accepts for the  property  argument.
   getCommonPropertyType(ELContext, Object)   - 
Method in class org.jdesktop.el. MapELResolver 
 If the base object is a map, returns the most general type that 
 this resolver accepts for the  property  argument.
   getCommonPropertyType(ELContext, Object)   - 
Method in class org.jdesktop.el. ResourceBundleELResolver 
 If the base object is a ResourceBundle, returns the most general type
 that this resolver accepts for the  property  argument.
   getContext(Class)   - 
Method in class org.jdesktop.el. ELContext 
 Returns the context object associated with the given key.
   getContext(Class)   - 
Method in class org.jdesktop.el.impl.lang. EvaluationContext 
  
   getConversionException()   - 
Method in class org.jdesktop.beansbinding. Binding.SyncFailure 
 Returns the exception that occurred during conversion if
 this failure represents a conversion failure.
   getConverter()   - 
Method in class org.jdesktop.beansbinding. Binding 
 Returns the  Binding's Converter , which may be  null .
   getDescription()   - 
Method in class org.jdesktop.beansbinding. Validator.Result 
 Returns a description of the validation result, which may be  null .
   getDetailBinding()   - 
Method in class org.jdesktop.swingbinding. JListBinding 
 Returns the  DetailBinding  for this  JListBinding .
   getELContext()   - 
Method in class org.jdesktop.el. ELContextEvent 
 Returns the  ELContext  that was created.
   getELContext()   - 
Method in class org.jdesktop.el.impl.lang. EvaluationContext 
  
   getElement(int)   - 
Method in class org.jdesktop.swingbinding.impl. ListBindingManager 
  
   getElements()   - 
Method in class org.jdesktop.swingbinding.impl. ListBindingManager 
  
   getELResolver()   - 
Method in class org.jdesktop.el. ELContext 
 Retrieves the  ELResolver  associated with this context.
   getELResolver()   - 
Method in class org.jdesktop.el.impl.lang. EvaluationContext 
  
   getEndColumn()   - 
Method in class org.jdesktop.el.impl.parser. SimpleCharStream 
  
   getEndLine()   - 
Method in class org.jdesktop.el.impl.parser. SimpleCharStream 
  
   getErrorCode()   - 
Method in class org.jdesktop.beansbinding. Validator.Result 
 Returns the error code for the result, which may be  null .
   getExpectedType()   - 
Method in class org.jdesktop.el.impl. ValueExpressionImpl 
  
   getExpectedType()   - 
Method in class org.jdesktop.el.impl. ValueExpressionLiteral 
  
   getExpectedType()   - 
Method in class org.jdesktop.el. ValueExpression 
 Returns the type the result of the expression will be coerced to 
 after evaluation.
   getExpression()   - 
Method in class org.jdesktop.el.impl.lang. EvaluationContext 
  
   getExpressionString()   - 
Method in class org.jdesktop.el. Expression 
 Returns the original String used to create this  Expression ,
 unmodified.
   getExpressionString()   - 
Method in class org.jdesktop.el.impl. MethodExpressionImpl 
 Returns the original String used to create this  Expression ,
 unmodified.
   getExpressionString()   - 
Method in class org.jdesktop.el.impl. MethodExpressionLiteral 
  
   getExpressionString()   - 
Method in class org.jdesktop.el.impl. ValueExpressionImpl 
 Returns the type the result of the expression will be coerced to after
 evaluation.
   getExpressionString()   - 
Method in class org.jdesktop.el.impl. ValueExpressionLiteral 
  
   getFailure()   - 
Method in class org.jdesktop.beansbinding. Binding.ValueResult 
 Returns the failure if this  ValueResult  represents
 a failure and throws  UnsupportedOperationException 
 otherwise.
   getFeatureDescriptors(ELContext, Object)   - 
Method in class org.jdesktop.el. ArrayELResolver 
 Always returns  null , since there is no reason to 
 iterate through set set of all integers.
   getFeatureDescriptors(ELContext, Object)   - 
Method in class org.jdesktop.el. BeanELResolver 
 If the base object is not  null , returns an
  Iterator  containing the set of JavaBeans properties
 available on the given object.
   getFeatureDescriptors(ELContext, Object)   - 
Method in class org.jdesktop.el. CompositeELResolver 
 Returns information about the set of variables or properties that 
 can be resolved for the given  base  object.
   getFeatureDescriptors(ELContext, Object)   - 
Method in class org.jdesktop.el. ELResolver 
 Returns information about the set of variables or properties that 
 can be resolved for the given  base  object.
   getFeatureDescriptors(ELContext, Object)   - 
Method in class org.jdesktop.el. ListELResolver 
 Always returns  null , since there is no reason to 
 iterate through set set of all integers.
   getFeatureDescriptors(ELContext, Object)   - 
Method in class org.jdesktop.el. MapELResolver 
 If the base object is a map, returns an  Iterator 
 containing the set of keys available in the  Map .
   getFeatureDescriptors(ELContext, Object)   - 
Method in class org.jdesktop.el. ResourceBundleELResolver 
 If the base object is a ResourceBundle, returns an  Iterator 
 containing the set of keys available in the  ResourceBundle .
   getFloatingPoint()   - 
Method in class org.jdesktop.el.impl.parser. AstFloatingPoint 
  
   getFunctionMapper()   - 
Method in class org.jdesktop.el. ELContext 
 Retrieves the  FunctionMapper  associated with this 
  ELContext .
   getFunctionMapper()   - 
Method in class org.jdesktop.el.impl.lang. EvaluationContext 
  
   getImage()   - 
Method in interface org.jdesktop.el.impl.parser. Node 
  
   GetImage()   - 
Method in class org.jdesktop.el.impl.parser. SimpleCharStream 
  
   getImage()   - 
Method in class org.jdesktop.el.impl.parser. SimpleNode 
  
   getInteger()   - 
Method in class org.jdesktop.el.impl.parser. AstInteger 
  
   getLine()   - 
Method in class org.jdesktop.el.impl.parser. SimpleCharStream 
  Deprecated.     
   getLocale()   - 
Method in class org.jdesktop.el. ELContext 
 Get the  Locale  stored by a previous invocation to 
   ELContext.setLocale(java.util.Locale)  .
   getLocalName()   - 
Method in class org.jdesktop.el.impl.parser. AstFunction 
  
   getMessage()   - 
Method in exception org.jdesktop.el.impl.parser. ParseException 
 This method has the standard behavior when this object has been
 created using the standard constructors.
   getMessage()   - 
Method in error org.jdesktop.el.impl.parser. TokenMgrError 
 You can also modify the body of this method to customize your error messages.
   getMethod()   - 
Method in class org.jdesktop.el.impl.lang. FunctionMapperImpl.Function 
  
   getMethod(Object, Object, Class[])   - 
Static method in class org.jdesktop.el.impl.util. ReflectionUtil 
 Returns a method based on the criteria
   getMethodInfo(ELContext)   - 
Method in class org.jdesktop.el.impl. MethodExpressionImpl 
 Evaluates the expression relative to the provided context, and returns
 information about the actual referenced method.
   getMethodInfo(ELContext)   - 
Method in class org.jdesktop.el.impl. MethodExpressionLiteral 
  
   getMethodInfo(EvaluationContext, Class[])   - 
Method in class org.jdesktop.el.impl.parser. AstIdentifier 
  
   getMethodInfo(EvaluationContext, Class[])   - 
Method in class org.jdesktop.el.impl.parser. AstValue 
  
   getMethodInfo(EvaluationContext, Class[])   - 
Method in interface org.jdesktop.el.impl.parser. Node 
  
   getMethodInfo(EvaluationContext, Class[])   - 
Method in class org.jdesktop.el.impl.parser. SimpleNode 
  
   getMethodInfo(ELContext)   - 
Method in class org.jdesktop.el. MethodExpression 
 Evaluates the expression relative to the provided context, and
 returns information about the actual referenced method.
   getName()   - 
Method in class org.jdesktop.beansbinding. Binding 
 Returns the  Binding's  name, which may be  null .
   getName()   - 
Method in class org.jdesktop.el. MethodInfo 
 Returns the name of the method
   getNewValue()   - 
Method in class org.jdesktop.beansbinding. PropertyStateEvent 
 Returns the new value of the  Property  for the source object,
 or  UNREADABLE  if the  Property  is not currently readable for the
 source object.
   getNextToken()   - 
Method in class org.jdesktop.el.impl.parser. ELParser 
  
   getNextToken()   - 
Method in class org.jdesktop.el.impl.parser. ELParserTokenManager 
  
   getObservableList()   - 
Method in class org.jdesktop.observablecollections. ObservableCollections.ObservableListHelper 
 Returns the  ObservableList .
   getOldValue()   - 
Method in class org.jdesktop.beansbinding. PropertyStateEvent 
 Returns the old value of the  Property  for the source object,
 or  UNREADABLE  if the  Property  was not previously readable for the
 source object.
   getOutputName()   - 
Method in class org.jdesktop.el.impl.parser. AstFunction 
  
   getParamTypes()   - 
Method in class org.jdesktop.el. MethodInfo 
 Returns the parameter types of the method
   getPrefix()   - 
Method in class org.jdesktop.el.impl.parser. AstFunction 
  
   getProperty()   - 
Method in class org.jdesktop.el. Expression.ResolvedProperty 
  
   getPropertyChangeListeners()   - 
Method in class org.jdesktop.beansbinding. Binding 
 Returns the list of  PropertyChangeListeners  registered on this
  Binding .
   getPropertyChangeListeners(String)   - 
Method in class org.jdesktop.beansbinding. Binding 
 Returns the list of  PropertyChangeListeners  registered on this
  Binding  for the given property name.
   getPropertyChangeListeners()   - 
Method in class org.jdesktop.swingbinding.adapters. BeanAdapterBase 
  
   getPropertyChangeListeners(String)   - 
Method in class org.jdesktop.swingbinding.adapters. BeanAdapterBase 
  
   getPropertyDescriptor(Object, Object)   - 
Static method in class org.jdesktop.el.impl.util. ReflectionUtil 
  
   getPropertyStateListeners(S)   - 
Method in class org.jdesktop.beansbinding. ObjectProperty 
 Returns an empty array; the state of an  ObjectProperty  never changes
 so listeners aren't useful.
   getPropertyStateListeners(S)   - 
Method in class org.jdesktop.beansbinding. Property 
 Returns an arry containing the listeners registered for the given source.
   getPropertyStateListeners(S)   - 
Method in class org.jdesktop.beansbinding. PropertyHelper 
 Returns an arry containing the listeners registered for the given source.
   getPropertyType()   - 
Method in class org.jdesktop.el. BeanELResolver.BeanProperty 
  
   getReadableChanged()   - 
Method in class org.jdesktop.beansbinding. PropertyStateEvent 
 Returns whether or not the  Property's  readability has changed for
 the source object.
   getReadMethod()   - 
Method in class org.jdesktop.el. BeanELResolver.BeanProperty 
  
   getResolvedProperties()   - 
Method in class org.jdesktop.el. Expression.Result 
  
   getResolvedProperties()   - 
Method in class org.jdesktop.el.impl.lang. EvaluationContext 
  
   getResult()   - 
Method in class org.jdesktop.el. Expression.Result 
  
   getResult(ELContext, boolean)   - 
Method in class org.jdesktop.el.impl. ValueExpressionImpl 
  
   getResult(ELContext, boolean)   - 
Method in class org.jdesktop.el. ValueExpression 
  
   getReturnType()   - 
Method in class org.jdesktop.el. MethodInfo 
 Returns the return type of the method
   getSelectedElement()   - 
Method in class org.jdesktop.swingbinding.adapters. JListAdapterProvider.Adapter 
  
   getSelectedElement()   - 
Method in class org.jdesktop.swingbinding.adapters. JTableAdapterProvider.Adapter 
  
   getSelectedElement_IGNORE_ADJUSTING()   - 
Method in class org.jdesktop.swingbinding.adapters. JListAdapterProvider.Adapter 
  
   getSelectedElement_IGNORE_ADJUSTING()   - 
Method in class org.jdesktop.swingbinding.adapters. JTableAdapterProvider.Adapter 
  
   getSelectedElements()   - 
Method in class org.jdesktop.swingbinding.adapters. JListAdapterProvider.Adapter 
  
   getSelectedElements()   - 
Method in class org.jdesktop.swingbinding.adapters. JTableAdapterProvider.Adapter 
  
   getSelectedElements_IGNORE_ADJUSTING()   - 
Method in class org.jdesktop.swingbinding.adapters. JListAdapterProvider.Adapter 
  
   getSelectedElements_IGNORE_ADJUSTING()   - 
Method in class org.jdesktop.swingbinding.adapters. JTableAdapterProvider.Adapter 
  
   getSelectedItem()   - 
Method in class org.jdesktop.swingbinding.adapters. JComboBoxAdapterProvider.Adapter 
  
   getSource()   - 
Method in class org.jdesktop.beansbinding. PropertyStateEvent 
 Returns the  Property  whose state has changed.
   getSource()   - 
Method in class org.jdesktop.el. Expression.ResolvedProperty 
  
   getSource()   - 
Method in class org.jdesktop.el. ValueExpression 
 Returns the source of the expression.
   getSourceNullValue()   - 
Method in class org.jdesktop.beansbinding. Binding 
 Returns the value to be returned by   Binding.getSourceValueForTarget()  
 when the source property returns  null  for the source object.
   getSourceObject()   - 
Method in class org.jdesktop.beansbinding. Binding 
 Returns the  Binding's  source object, which may be  null .
   getSourceObject()   - 
Method in class org.jdesktop.beansbinding. PropertyStateEvent 
 Returns the source object for which the  Property's  state has changed.
   getSourceProperty()   - 
Method in class org.jdesktop.beansbinding. Binding 
 Returns the  Binding's  source property, which may not be  null .
   getSourceProperty()   - 
Method in class org.jdesktop.beansbinding. PropertyStateEvent 
 Returns the  Property  whose state has changed.
   getSourceUnreadableValue()   - 
Method in class org.jdesktop.beansbinding. Binding 
 If set, returns the value to be returned by   Binding.getSourceValueForTarget()  
 when the source property is unreadable for the source object.
   getSourceValueForTarget()   - 
Method in class org.jdesktop.beansbinding. Binding 
 Fetches the value of the source property for the source object and
 returns a  ValueResult  representing that value in terms that
 can be set on the target property for the target object.
   getString()   - 
Method in class org.jdesktop.el.impl.parser. AstString 
  
   GetSuffix(int)   - 
Method in class org.jdesktop.el.impl.parser. SimpleCharStream 
  
   getTargetNullValue()   - 
Method in class org.jdesktop.beansbinding. Binding 
 Returns the value to be returned by   Binding.getTargetValueForSource()  
 when the target property returns  null  for the target object.
   getTargetObject()   - 
Method in class org.jdesktop.beansbinding. Binding 
 Returns the  Binding's  target object, which may be  null .
   getTargetProperty()   - 
Method in class org.jdesktop.beansbinding. Binding 
 Returns the  Binding's  target property, which may not be  null .
   getTargetValueForSource()   - 
Method in class org.jdesktop.beansbinding. Binding 
 Fetches the value of the target property for the target object and
 returns a  ValueResult  representing that value in terms that
 can be set on the source property for the source object.
   getText()   - 
Method in class org.jdesktop.swingbinding.adapters. JTextComponentAdapterProvider.Adapter 
  
   getText_ON_ACTION_OR_FOCUS_LOST()   - 
Method in class org.jdesktop.swingbinding.adapters. JTextComponentAdapterProvider.Adapter 
  
   getText_ON_FOCUS_LOST()   - 
Method in class org.jdesktop.swingbinding.adapters. JTextComponentAdapterProvider.Adapter 
  
   getToken(int)   - 
Method in class org.jdesktop.el.impl.parser. ELParser 
  
   getType()   - 
Method in class org.jdesktop.beansbinding. Binding.SyncFailure 
 Returns the type of failure.
   getType(ELContext, Object, Object)   - 
Method in class org.jdesktop.el. ArrayELResolver 
 If the base object is an array, returns the most general acceptable type 
 for a value in this array.
   getType(ELContext, Object, Object)   - 
Method in class org.jdesktop.el. BeanELResolver 
 If the base object is not  null , returns the most 
 general acceptable type that can be set on this bean property.
   getType(ELContext, Object, Object)   - 
Method in class org.jdesktop.el. CompositeELResolver 
 For a given  base  and  property , attempts to
 identify the most general type that is acceptable for an object to be 
 passed as the  value  parameter in a future call 
 to the   CompositeELResolver.setValue(org.jdesktop.el.ELContext, java.lang.Object, java.lang.Object, java.lang.Object)   method.
   getType(ELContext, Object, Object)   - 
Method in class org.jdesktop.el. ELResolver 
 For a given  base  and  property , attempts to
 identify the most general type that is acceptable for an object to be 
 passed as the  value  parameter in a future call 
 to the   ELResolver.setValue(org.jdesktop.el.ELContext, java.lang.Object, java.lang.Object, java.lang.Object)   method.
   getType()   - 
Method in class org.jdesktop.el. Expression.Result 
  
   getType(EvaluationContext)   - 
Method in class org.jdesktop.el.impl.parser. ArithmeticNode 
  
   getType(EvaluationContext)   - 
Method in class org.jdesktop.el.impl.parser. AstChoice 
  
   getType(EvaluationContext)   - 
Method in class org.jdesktop.el.impl.parser. AstCompositeExpression 
  
   getType(EvaluationContext)   - 
Method in class org.jdesktop.el.impl.parser. AstDeferredExpression 
  
   getType(EvaluationContext)   - 
Method in class org.jdesktop.el.impl.parser. AstDynamicExpression 
  
   getType(EvaluationContext)   - 
Method in class org.jdesktop.el.impl.parser. AstEmpty 
  
   getType(EvaluationContext)   - 
Method in class org.jdesktop.el.impl.parser. AstFloatingPoint 
  
   getType(EvaluationContext)   - 
Method in class org.jdesktop.el.impl.parser. AstFunction 
  
   getType(EvaluationContext)   - 
Method in class org.jdesktop.el.impl.parser. AstIdentifier 
  
   getType(EvaluationContext)   - 
Method in class org.jdesktop.el.impl.parser. AstInteger 
  
   getType(EvaluationContext)   - 
Method in class org.jdesktop.el.impl.parser. AstLiteralExpression 
  
   getType(EvaluationContext)   - 
Method in class org.jdesktop.el.impl.parser. AstNegative 
  
   getType(EvaluationContext)   - 
Method in class org.jdesktop.el.impl.parser. AstNot 
  
   getType(EvaluationContext)   - 
Method in class org.jdesktop.el.impl.parser. AstNull 
  
   getType(EvaluationContext)   - 
Method in class org.jdesktop.el.impl.parser. AstString 
  
   getType(EvaluationContext)   - 
Method in class org.jdesktop.el.impl.parser. AstValue 
  
   getType(EvaluationContext)   - 
Method in class org.jdesktop.el.impl.parser. BooleanNode 
  
   getType(EvaluationContext)   - 
Method in interface org.jdesktop.el.impl.parser. Node 
  
   getType(EvaluationContext)   - 
Method in class org.jdesktop.el.impl.parser. SimpleNode 
  
   getType(ELContext)   - 
Method in class org.jdesktop.el.impl. ValueExpressionImpl 
  
   getType(ELContext)   - 
Method in class org.jdesktop.el.impl. ValueExpressionLiteral 
  
   getType(ELContext, Object, Object)   - 
Method in class org.jdesktop.el. ListELResolver 
 If the base object is a list, returns the most general acceptable type 
 for a value in this list.
   getType(ELContext, Object, Object)   - 
Method in class org.jdesktop.el. MapELResolver 
 If the base object is a map, returns the most general acceptable type 
 for a value in this map.
   getType(ELContext, Object, Object)   - 
Method in class org.jdesktop.el. ResourceBundleELResolver 
 If the base object is an instance of  ResourceBundle ,
 return  null , since the resolver is read only.
   getType(ELContext)   - 
Method in class org.jdesktop.el. ValueExpression 
 Evaluates the expression relative to the provided context, and 
 returns the most general type that is acceptable for an object to be 
 passed as the  value  parameter in a future call 
 to the   ValueExpression.setValue(org.jdesktop.el.ELContext, java.lang.Object)   method.
   getUpdateStrategy()   - 
Method in class org.jdesktop.beansbinding. AutoBinding 
 Returns the  AutoBinding's  update strategy.
   getValidationResult()   - 
Method in class org.jdesktop.beansbinding. Binding.SyncFailure 
 Returns the result that was returned from the
  Binding's  validator if this failure represents a
 validation failure.
   getValidator()   - 
Method in class org.jdesktop.beansbinding. Binding 
 Returns the  Binding's Validator , which may be  null .
   getValue(S)   - 
Method in class org.jdesktop.beansbinding. BeanProperty 
 Returns the value of this  Property  for the given source.
   getValue()   - 
Method in class org.jdesktop.beansbinding. Binding.ValueResult 
 Returns the resulting value if this  ValueResult  does
 not represent a failure and throws  UnsupportedOperationException 
 otherwise.
   getValue(S)   - 
Method in class org.jdesktop.beansbinding. ELProperty 
 Returns the value of this  Property  for the given source.
   getValue(S)   - 
Method in class org.jdesktop.beansbinding. ObjectProperty 
 Returns the source object passed to the method.
   getValue(S)   - 
Method in class org.jdesktop.beansbinding. Property 
 Returns the value of this  Property  for the given source.
   getValue(S)   - 
Method in class org.jdesktop.beansbinding. PropertyHelper 
 Returns the value of this  Property  for the given source.
   getValue(ELContext, Object, Object)   - 
Method in class org.jdesktop.el. ArrayELResolver 
 If the base object is a Java language array, returns the value at the 
 given index.
   getValue(ELContext, Object, Object)   - 
Method in class org.jdesktop.el. BeanELResolver 
 If the base object is not  null , returns the current
 value of the given property on this bean.
   getValue(ELContext, Object, Object)   - 
Method in class org.jdesktop.el. CompositeELResolver 
 Attempts to resolve the given  property  object on the given
  base  object by querying all component resolvers.
   getValue(ELContext, Object, Object)   - 
Method in class org.jdesktop.el. ELResolver 
 Attempts to resolve the given  property  object on the given
  base  object.
   getValue(EvaluationContext)   - 
Method in class org.jdesktop.el.impl.parser. AstAnd 
  
   getValue(EvaluationContext)   - 
Method in class org.jdesktop.el.impl.parser. AstBracketSuffix 
  
   getValue(EvaluationContext)   - 
Method in class org.jdesktop.el.impl.parser. AstChoice 
  
   getValue(EvaluationContext)   - 
Method in class org.jdesktop.el.impl.parser. AstCompositeExpression 
  
   getValue(EvaluationContext)   - 
Method in class org.jdesktop.el.impl.parser. AstDeferredExpression 
  
   getValue(EvaluationContext)   - 
Method in class org.jdesktop.el.impl.parser. AstDiv 
  
   getValue(EvaluationContext)   - 
Method in class org.jdesktop.el.impl.parser. AstDotSuffix 
  
   getValue(EvaluationContext)   - 
Method in class org.jdesktop.el.impl.parser. AstDynamicExpression 
  
   getValue(EvaluationContext)   - 
Method in class org.jdesktop.el.impl.parser. AstEmpty 
  
   getValue(EvaluationContext)   - 
Method in class org.jdesktop.el.impl.parser. AstEqual 
  
   getValue(EvaluationContext)   - 
Method in class org.jdesktop.el.impl.parser. AstFalse 
  
   getValue(EvaluationContext)   - 
Method in class org.jdesktop.el.impl.parser. AstFloatingPoint 
  
   getValue(EvaluationContext)   - 
Method in class org.jdesktop.el.impl.parser. AstFunction 
  
   getValue(EvaluationContext)   - 
Method in class org.jdesktop.el.impl.parser. AstGreaterThan 
  
   getValue(EvaluationContext)   - 
Method in class org.jdesktop.el.impl.parser. AstGreaterThanEqual 
  
   getValue(EvaluationContext)   - 
Method in class org.jdesktop.el.impl.parser. AstIdentifier 
  
   getValue(EvaluationContext)   - 
Method in class org.jdesktop.el.impl.parser. AstInteger 
  
   getValue(EvaluationContext)   - 
Method in class org.jdesktop.el.impl.parser. AstLessThan 
  
   getValue(EvaluationContext)   - 
Method in class org.jdesktop.el.impl.parser. AstLessThanEqual 
  
   getValue(EvaluationContext)   - 
Method in class org.jdesktop.el.impl.parser. AstLiteralExpression 
  
   getValue(EvaluationContext)   - 
Method in class org.jdesktop.el.impl.parser. AstMinus 
  
   getValue(EvaluationContext)   - 
Method in class org.jdesktop.el.impl.parser. AstMod 
  
   getValue(EvaluationContext)   - 
Method in class org.jdesktop.el.impl.parser. AstMult 
  
   getValue(EvaluationContext)   - 
Method in class org.jdesktop.el.impl.parser. AstNegative 
  
   getValue(EvaluationContext)   - 
Method in class org.jdesktop.el.impl.parser. AstNot 
  
   getValue(EvaluationContext)   - 
Method in class org.jdesktop.el.impl.parser. AstNotEqual 
  
   getValue(EvaluationContext)   - 
Method in class org.jdesktop.el.impl.parser. AstNull 
  
   getValue(EvaluationContext)   - 
Method in class org.jdesktop.el.impl.parser. AstOr 
  
   getValue(EvaluationContext)   - 
Method in class org.jdesktop.el.impl.parser. AstPlus 
  
   getValue(EvaluationContext)   - 
Method in class org.jdesktop.el.impl.parser. AstString 
  
   getValue(EvaluationContext)   - 
Method in class org.jdesktop.el.impl.parser. AstTrue 
  
   getValue(EvaluationContext)   - 
Method in class org.jdesktop.el.impl.parser. AstValue 
  
   getValue(EvaluationContext)   - 
Method in interface org.jdesktop.el.impl.parser. Node 
  
   getValue(EvaluationContext)   - 
Method in class org.jdesktop.el.impl.parser. SimpleNode 
  
   getValue(ELContext)   - 
Method in class org.jdesktop.el.impl. ValueExpressionImpl 
  
   getValue(ELContext)   - 
Method in class org.jdesktop.el.impl. ValueExpressionLiteral 
  
   getValue(ELContext, Object, Object)   - 
Method in class org.jdesktop.el. ListELResolver 
 If the base object is a list, returns the value at the given index.
   getValue(ELContext, Object, Object)   - 
Method in class org.jdesktop.el. MapELResolver 
 If the base object is a map, returns the value associated with the
 given key, as specified by the  property  argument.
   getValue(ELContext, Object, Object)   - 
Method in class org.jdesktop.el. ResourceBundleELResolver 
 If the base object is an instance of  ResourceBundle ,
 the provided property will first be coerced to a  String .
   getValue(ELContext)   - 
Method in class org.jdesktop.el. ValueExpression 
 Evaluates the expression relative to the provided context, and 
 returns the resulting value.
   getValue()   - 
Method in class org.jdesktop.swingbinding.adapters. JSliderAdapterProvider.Adapter 
  
   getValue()   - 
Method in class org.jdesktop.swingbinding.adapters. JSpinnerAdapterProvider.Adapter 
  
   getValue_IGNORE_ADJUSTING()   - 
Method in class org.jdesktop.swingbinding.adapters. JSliderAdapterProvider.Adapter 
  
   getValueChanged()   - 
Method in class org.jdesktop.beansbinding. PropertyStateEvent 
 Returns whether or not the  Property's  value has changed for the source object.
   getVariableMapper()   - 
Method in class org.jdesktop.el. ELContext 
 Retrieves the  VariableMapper  associated with this 
  ELContext .
   getVariableMapper()   - 
Method in class org.jdesktop.el.impl.lang. EvaluationContext 
  
   getWriteableChanged()   - 
Method in class org.jdesktop.beansbinding. PropertyStateEvent 
 Returns whether or not the  Property's  writeability has changed for
 the source object.
   getWriteMethod()   - 
Method in class org.jdesktop.el. BeanELResolver.BeanProperty 
  
   getWriteType(S)   - 
Method in class org.jdesktop.beansbinding. BeanProperty 
 Returns the type of object that is suitable for setting as the value
 of this  Property  by calls to  setValue .
   getWriteType(S)   - 
Method in class org.jdesktop.beansbinding. ELProperty 
 Returns the type of object that is suitable for setting as the value
 of this  Property  by calls to  setValue .
   getWriteType(S)   - 
Method in class org.jdesktop.beansbinding. ObjectProperty 
 Throws  UnsupportedOperationException ;  ObjectProperty  is never writeable.
   getWriteType(S)   - 
Method in class org.jdesktop.beansbinding. Property 
 Returns the type of object that is suitable for setting as the value
 of this  Property  by calls to  setValue .
   getWriteType(S)   - 
Method in class org.jdesktop.beansbinding. PropertyHelper 
 Returns the type of object that is suitable for setting as the value
 of this  Property  by calls to  setValue .
   GT0   - 
Static variable in interface org.jdesktop.el.impl.parser. ELParserConstants 
  
   GT1   - 
Static variable in interface org.jdesktop.el.impl.parser. ELParserConstants 
  
 
 


 
   
  
 
 
 
   
 
   
          Overview     
        Package   
        Class   
        Use   
          Tree     
          Deprecated     
       Index    
          Help     
   
 
 
  
 
 
 

 
  
   PREV LETTER   
   NEXT LETTER    
  
    FRAMES     
   NO FRAMES     
  
    All Classes  ');
  }
  //-->
 
 
    All Classes  
 


  
 
 
  
 

 A   B   C   D   E   F   G   H   I   J   K   L   M   N   O   P   Q   R   S   T   U   V   W
